# Snail1 expression in colorectal cancer and its correlation with clinical and pathological parameters

**DOI:** 10.1186/1471-2407-13-145

**Published:** 2013-03-22

**Authors:** Feride Kroepil, Georg Fluegen, Daniel Vallböhmer, Stephan E Baldus, Levent Dizdar, Andreas M Raffel, Dieter Hafner, Nikolas H Stoecklein, Wolfram T Knoefel

**Affiliations:** 1 Department of Surgery (A), Heinrich-Heine-University and University Hospital Duesseldorf, Düsseldorf, 40225, Germany; 2 Institute for Pathology, University Hospital Düsseldorf, Düsseldorf, Germany; 3 Institute for Pharmacology, Heinrich Heine University of Düsseldorf, Düsseldorf, Germany

**Keywords:** Snail1, E-cadherin, Colorectal carcinoma, Prognostic factor, EMT

## Abstract

**Background:**

Snail1 is a transcription regulator of E-cadherin. The loss of E-cadherin seems to be a crucial step in the process of Epithelial-mesenchymal transition (EMT). EMT initiates invasion and proliferation in many tumours. Overexpression of Snail1 is known to be associated with poor outcome in several solid tumours. The aim of this study was to analyse its expression profile and prognostic significance in colorectal cancer.

**Methods:**

Tissue microarrays (TMA) containing paraffin-embedded primary colorectal cancer (CRC) tissue samples from 251 patients were used in this study. The expression of Snail1 and E-cadherin was assessed by immunohistochemistry in different tumour compartments, corresponding lymph node metastases and normal colonic mucosa. Intensity of staining was classified according to the Remmele score (standardized scoring system) as well as the semiquantitative score established by Blechschmidt et al.

**Results:**

Snail1 expression was observed in 76% of the CRC. Loss of E-cadherin was noted in 87% of the CRC. Snail1 positive tumours were significantly correlated with Snail1 positive lymph node metastases (p=0.03). There was no significant correlation between loss of E-cadherin and Snail1 expression, or between N-stage or grading and Snail1 expression. Kaplan-Meier survival analysis identified no prognostic impact of Snail1 expression on overall survival.

**Conclusion:**

Snail1 expression was detectable in most of the CRC but showed no significant association with E-cadherin loss, clinical pathological characteristics or overall survival. The observed loss of E-cadherin could be explained by effects of other important EMT pathways, such as the Wnt-signalling cascade.

## Background

Snail1 was the first characterized repressor of the invasion suppressor gene CDH1, which encodes for the crucial adhesion protein E-cadherin [1,2]. Snail1 can bind to specific E-box regions on the CDH1 promotor, thus leading to transcriptional repression of E-cadherin. E-cadherin is a member of a family of transmembrane glycoproteins that mediate intercellular adhesion [3]. Loss of its expression or function diminishes cell–cell contacts and is known to be a key step during the process of Epithelial-mesenchymal transition (EMT). EMT describes a phenotypic change in cells from epithelial to mesenchymal properties. By activating this process epithelial cells can dispose of their differentiated characteristics and gain mesenchymal features such as invasiveness, motility and increased apoptotic resistance [4]. This reversible EMT process is crucial in embryonic development for the correct implantation of the embryo and during gastrulation and organogenesis [5,6]. In differentiated somatic cells this programme of EMT is normally inactive [6]. Reactivation of this programme is known to be a crucial event in tumour progression. During this process, cancer cells change their phenotype from epithelial to mesenchymal and gain the ability to invade and metastasize. E-cadherin expression is frequently downregulated in many different types of tumour, where it accompanies the invasiveness and metastatic behaviour of malignant cells [6,7].

Besides their involvement in EMT, Snail family members are involved in a variety of other processes, such as apoptosis or mesoderm formation in the developing embryo. Snail1 has recently been shown to activate Wnt/beta-Catenin signalling and nuclear factor kappa B activity [8,9], and it abrogates the inhibition of the Wnt/beta-Catenin pathway caused by the anti-tumoural compound 1a,25-dihydroxyvitamin D3 [10]. In several entities of human cancer, including skin [11], oral [12], breast [13], hepatocellular [14], gastric [15] and colon carcinomas [16], Snail1 is upregulated and frequently associated with invasiveness, metastases and poor prognosis [17,18]. The mechanism by which Snail1 influences these different cellular processes is still not totally understood.

Snail1 RNA is not detectable in normal colon mucosa, but is upregulated in 60–70% of colorectal adenoma and colorectal cancers (CRC) [16,19-21]. Importantly, aberrant Snail1 expression in CRC was associated not only with poor prognosis, but also with shortened relapse-free survival [20,22]. The tumour microenvironment, especially at the invasive front, is important for the formation of tumour buds in CRC. At the invasive front of CRC, the existence of tumour budding (TB: the detachment and migration of small clusters of tumour cells from the neoplastic epithelium) is correlated with a high incidence of local invasion and distant metastasis. In a recently published study of stage II CRC tissues, TB was associated with increased levels of Snail1 expression as well as a high incidence of metachronous lymph node metastasis. Interestingly, treatment with recombinant TGF-β1 increased the number of cells expressing CD133 and Snail1 [23].

Despite the fact that many valuable studies concerning Snail1 expression in CRC have been published, its incidence and its prognostic significance in colorectal cancer remain undetermined.

In order to investigate the expression profile of Snail1 in CRC, we assessed its expression in formalin-fixed and paraffin-embedded (FFPE) tissue samples of 251 patients. We tested the association between the expression of Snail1 and E-cadherin. Furthermore, different tumour compartments (tumour centre and invasion front) and histopathological as well as clinical aspects were considered.

## Methods

### Tissue samples and data acquisition

Paraffin-embedded tissue samples of 251 patients with CRC were obtained from the Institute of Pathology for immunohistochemical analysis. The specimens were previously fixed in 10% formaldehyde, according to established methods [24]. All tissues were verified and graded in the pathology department. Tumour grading was performed according to World Health Organization (WHO) standards. The samples were randomly selected by experienced pathologists (S.E.B) from the archives of the Department of Pathology of the University Hospital Duesseldorf based on the availability of follow-up data. All patients underwent curative surgery at the University Hospital Duesseldorf between 1996 and 2005. Patients with neoadjuvant therapy, extended lymphatic dissemination (N3), distant metastasis (M+) or incomplete resection (R1, R2) were excluded from the cohort. Overall survival data were retrieved from a prospectively maintained clinical database at our hospital.

### Ethics statement

The study was approved by the Ethics Committee of the Medical Faculty of the Heinrich-Heine University Düsseldorf.

### Tissue microarrays

Fourteen tissue microarrays (TMA) were used in this study. The TMAs contained paraffin-embedded primary CRC tissue, lymph node metastases and normal colonic tissue samples from archival patient specimens. Up to six cylinders of 1.0 mm diameter (two from cancer invasion front, two from inner tumour mass, one from normal tissue and one from lymph node metastases, if present) were taken from representative areas of donor blocks of each patient and transferred to paraffin recipient blocks, with 0.5 mm between each cylinder.

The clinicohistopathological characteristics of the colorectal cancer patients, including age at diagnosis, tumour stage, and histopathological grading, are summarized in Table 1. The difference in sample numbers between Snail1 and E-cadherin staining (251 vs. 250) is due to loss of one sample during staining.

**Table 1:** Characteristics of the TMA collective

		**Patients**		**Snail positive**	**E-Cadherin positive**
**Tissue:**					
	Tumor	251		76%	61%
	Lymphnode	47		70%	48%
**Tumorstage:**					
**T**	1	8/251	(3%)	75%	50%
	2	64/251	(26%)	84%	67%
	3	153/251	(61%)	74%	60%
	4	26/251	(10%)	69%	58%
**N**	0	146/251	(58%)	80%	64%
	1	64/251	(26%)	70%	59%
	2	41/251	(16%)	76%	54%
**M**	0	251/251	(100%)	76%	61%
	1	0/251	(0%)	0%	0%
**G**	1	2/251	(1%)	100%	50%
	2	209/251	(83%)	75%	64%
	3	40/251	(16%)	83%	50%
**Sex:**					
	female	103/251	(41%)	75%	52%
	male	148/251	(59%)	77%	67%
**Age** at diagnosis:					
	≤ 65 y	80/251	(32%)	78%	60%
	> 65 y	171/251	(68%)	75%	62%

### Immunohistochemistry

Serial 4 μm sections of TMA blocks were prepared on a microtome (Leica SM2000R).

For immunostaining, the slides were deparaffinised and epitopes were retrieved using Dako Retrieval Solution (Dako Cytomation, USA) at 95°C for 30 min, followed by cooling to room temperature for 20 min. Endogenous peroxidase was inactivated using 0.3% H_2_O_2_ for 30 min at room temperature. Subsequently, the sections were rinsed twice in phosphate buffered saline (PBS, pH 7.4) for 5 min. Immunostaining was performed with antibodies directed against E-cadherin (mouse monoclonal, 2 μg/ml) and Snail1 (rabbit polyclonal, 1 μg/ml). See Table 2. Incubation with the primary antibodies was performed in a moist chamber at room temperature for 30 min. The Vectastain ABC peroxidase kit was used according to the manufacturer’s instructions (Vector Lab, USA) for specific antibody binding. Isotype controls using MOPC-21 (mouse IgG1, 2 μg/ml) and X0903 (rabbit immunoglobulin fraction, 1 μg/ml) were carried out on serial sections of each sample. Diaminobenzidine (Liquid DAB, Dako Cytomation, USA) was used to stain the bound immunocomplex. All specimens were counterstained with haematoxylin and eosin. A semiquantitative evaluation was performed by two independent researchers using a Zeiss Axioskope.

**Table 2:** Concentration and supplier of the antibodies

**Antibody**	**Concentration**	**Supplier**
E cadherin (NCH-38)	2 μg/ml	DAKO
Snail1 (Ab17732)	1 μg/ml	AbCam
Mouse lgG1 (MOPC-21)	2 μg/ml	Sigma
Rabbit-lgG (X0903)	1 μg/ml	DAKO

### Evaluation of immunostaining

The sections were examined by two independent researchers. Tissue samples from spleen and placenta embedded in the TMA were used as an internal control of staining efficiency and evaluation. Immunohistochemical results were evaluated for nuclear (Snail1) and membrane (E-cadherin) -specific staining only.

For E-cadherin and Snail1 an immunoreactive score (IRS) was set up, following Remmele et al. [25]. The level of staining intensity (SI) was subdivided into four groups: 0 (negative), 1 (weak), 2 (moderate) and 3 (strong). The percentage of positive cells (PP) was regarded as 0 (none), 1 (≤10%), 2 (11–50%), 3 (51–80%) and 4 (>80% positive tumour cells). The product of SI and PP is the IRS (0–12). A score of 0–2 was regarded as negative, 3–12 as positive [25].

To compare the E-cadherin staining to the normal mucosa, we also used the semiquantitative score established by Blechschmidt et al. for the same purpose [26]. The level of staining intensity was again subdivided into groups ranging from 0–3. Tumours with less than 20% of E-cadherin positive cells in category 3 were regarded as downregulated compared to normal colonic mucosa.

### Statistical analysis

Statistical analysis was performed using the SPSS software (Version 18). The threshold for statistical significance was p<0.05. To compare two independent, non-parametric samples we used the Mann–Whitney-U test. All survival analyses were performed using the Kaplan-Meier method. The significance of differences between groups was assessed using the log rank test. The Cox-Regression analysis was used to evaluate the risk of differences between groups in the Kaplan-Meier survival analyses (hazard ratio).

In all boxplots, the boxed area corresponds to the 25th to 75th percentile. The horizontal bars indicate the median. The whiskers show the 5th to 95th percentile. All outliers are indicated as dots.

## Results

### Snail1 expression and its association with E-cadherin in colorectal cancer

**Figure 1:**
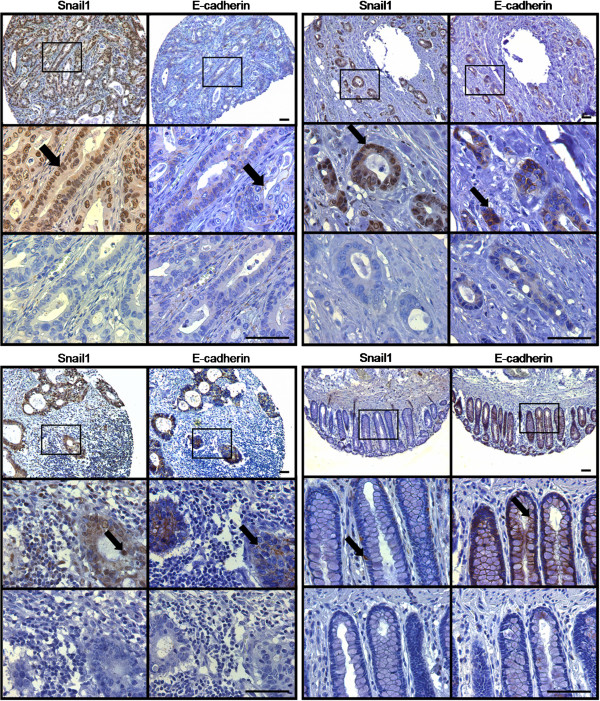
**Snail1 and E-cadherin staining.** Upper left: tumor center; upper right: invasion front; lower left: lymph node metastasis; lower right: normal colonic mucosa. Top row of each sample: positive staining (100x); middle row: detail of boxed area (400x), arrow points to positive nuclear (Snail1) or membranous (E-cadherin) staining; lower row: negative control of same area (400x). Scale = 100μm.

**Figure 2:**
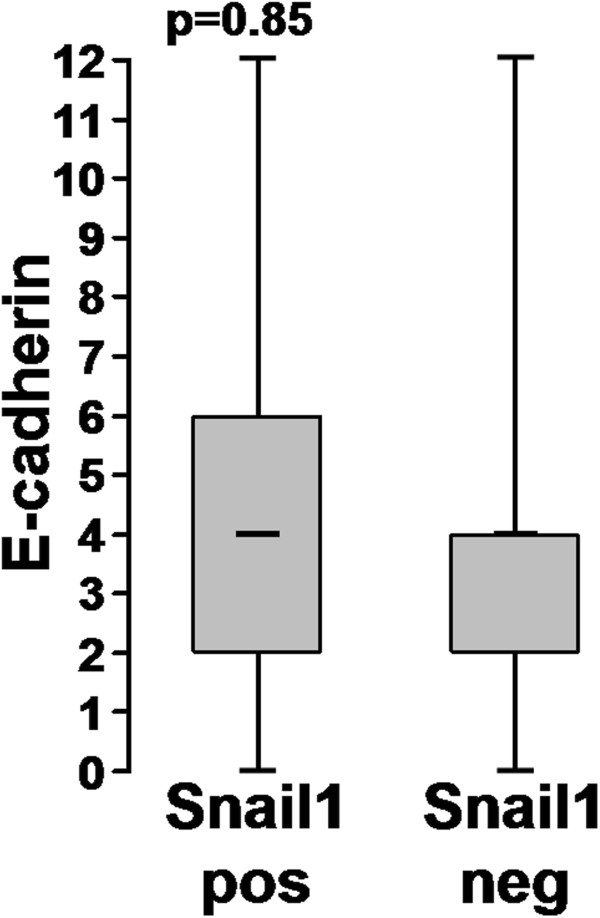
E-cadherin staining according to the Remmele score (y-axis) of Snail1 positive and negative tumors (x-axis).

**Figure 3:**
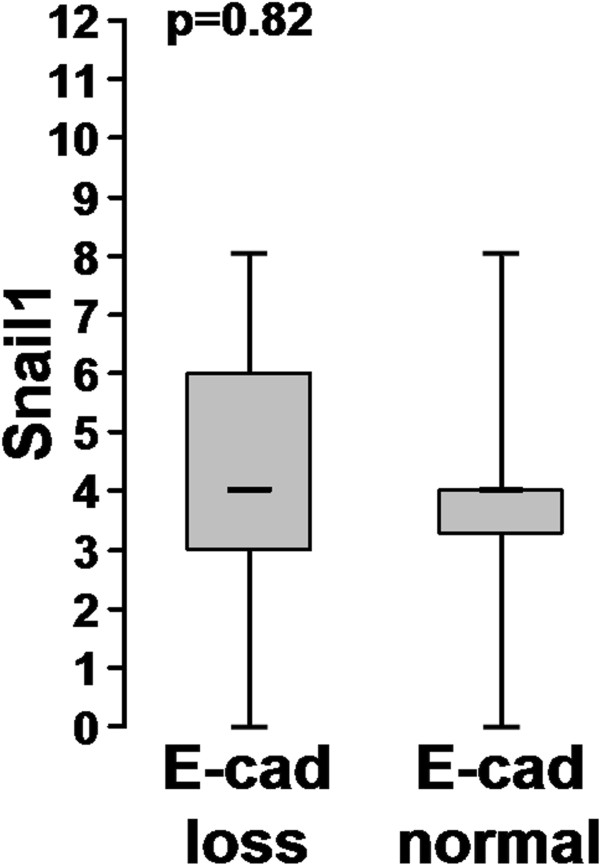
Snail1 staining according to the Remmele score (y-axis) of E-cadherin reduced or normal tumors (x-axis, Blechschmidt score: E-cadherin lost or normal compared to normal colonic mucosa).

We detected Snail1 in 76% (191/251) of the 251 samples, while E-cadherin expression was lacking in 39% (97/250) (Remmele score, Figure 1). In 87% (217/250), E-cadherin expression was downregulated compared to normal mucosa (Blechschmidt score [26]). We did not detect any correlation in the whole tumour between the expression of Snail1 and loss (Remmele score) (p=0.85) or even downregulation (Blechschmidt score) (p=0.82) of E-cadherin (Figures 2, 3). We detected no significant difference in the distribution of E-cadherin in the different tumour compartments (tumour centre, invasion front) and expression of Snail1 in the same compartment.

However, Snail1 positive tumours were significantly correlated with Snail1 positive lymph node metastases (p=0.03); but in those there was again no significant correlation between Snail1 and loss of E-cadherin (p=0.53).

### Snail1 and TNM

**Figure 4:**
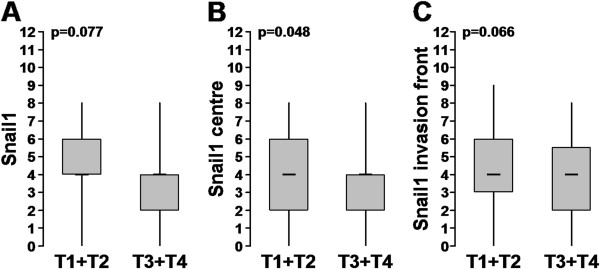
**A-C: Different Snail1 staining in small (T1+T2) and advanced (T3+T4) colorectal cancers.** Y-axis: Snail1 Remmele score. **A**: overall tumor; **B**: Snail1 staining in the tumor center; **C**: Snial1 staining in the invasion front.

Small tumours (T1+T2) showed a trend towards higher Snail1 expression, compared to advanced tumours (T3+T4) (p=0.077, Figure 4A). Although this correlation did not reach significance, when considering the separate compartments, we observed significantly higher expression of Snail1 in the tumour centre of small tumours (p=0.048, Figure 4B). Snail1 expression at the invasion front did not differ significantly between small and advanced tumours (p=0.066, Figure 4C).

Likewise, there was no difference in Snail1 expression between the different N-stages or between low-grade (G1+G2) and high-grade (G3+G4) cancer (p=0.42; p=0.17, respectively).

### E-cadherin and TNM

**Figure 5:**
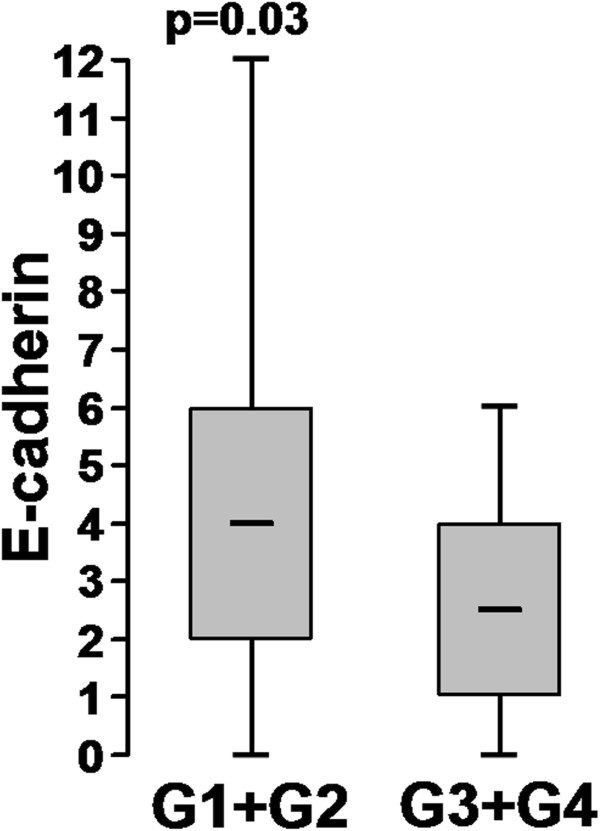
**Different E-cadherin staining in low grade (G1+G2) and high grade (G3+G4) tumors.** Y-axis: E-cadherin Remmele score.

There was a significant difference in E-cadherin expression between low-grade (G1+G2) and high-grade (G3+G4) CRC. The high-grade tumours showed significantly reduced E-cadherin expression (p=0.03, Figure 5).

In terms of T-stage, there was no correlation between E-cadherin and small or advanced tumours (p=0.17).

### Influence on overall survival

**Figure 6:**
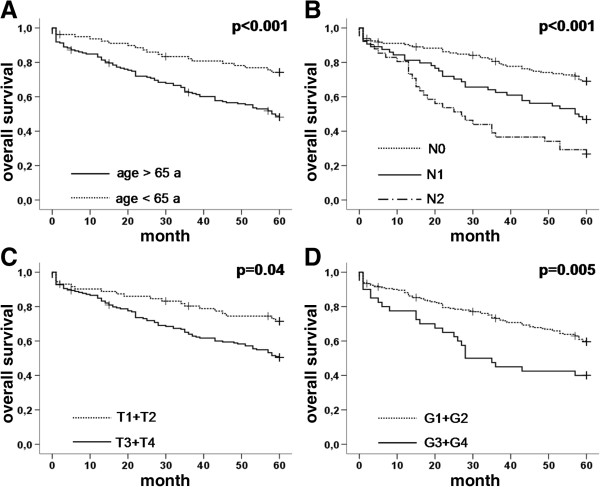
**Kaplan-Meier graphs showing the overall survival for A: age at diagnosis, B: N-stage, C: T-stage and D: grading.** All factors showed a significant impact on overall survival.

**Figure 7:**
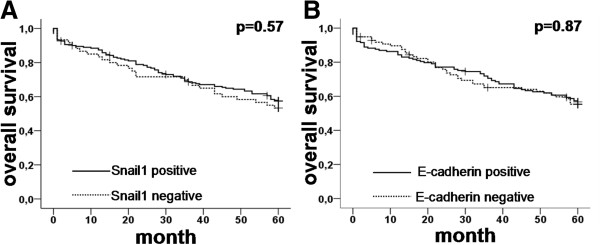
**Kaplan-Meier graphs showing the overall survival for A: Snail1 status and B: E-cadherin status.** Neither one showed any significant impact on overall survival.

Age at diagnosis, lymph node-metastasis (N), tumour stage (T) and grading (G) showed a significant influence on overall survival (Figure 6), while neither Snail1 nor E-cadherin expression seemed to have any effect (Figure 7). Cox-regression analysis of overall survival showed a hazard ratio (HR) of 1.7 for lymph node metastases, 1.6 for grading and 3.3 for age at diagnosis (p<0.0001, p=0.045, p<0.0001, respectively). There was no significant correlation between T-stage or sex and overall survival (p=0.1, p=1.0, respectively, Table 3).

**Table 3:** Cox-Regression of clinical parameters

**COX-Reg**	**stand. error**	**P value**	**HR**
sex	0.19	0.98	1,00
age	0.25	<0.0001	3,32
T	0.18	0.12	1,32
N	0.12	<0.0001	1,74
G	0.22	0.04	1,56

Stand. error = standard error; HR = hazard ratio.

### Effect of Snail1 and E-cadherin on UICC stage and tumour location

We found a significant correlation between UICC stage and tumour location (p=0.01). Tumours with a high UICC stage tended to be located in the right colon, while carcinomas of the left colon showed a lower UICC stage. The UICC stage showed no correlation with either E-cadherin or Snail1 expression. We were also unable to detect any difference in the expression of Snail1 or E-cadherin between rectal, left or right colon cancers.

## Discussion

Invasion and metastasis are life-limiting aspects of malignant tumours. It has been shown in a variety of studies that cancer cells use EMT to downregulate their cell–cell contacts and become motile and invasive [19]. Many authors regard EMT as a major mechanism enabling metastasis and initiating the transition between benign and malignant tissue.

Here, we analysed the nuclear expression of Snail1 transcriptional factor in a large cohort of human colorectal carcinomas. Snail1 is one of the best-characterized E-cadherin gene repressors required for triggering EMT. Only cells presenting immunostaining in the nucleus were considered Snail1-positive. The diffuse staining detected occasionally in the cytosol in some epithelial cells was not considered to indicate Snail1 expression, since Snail1 is not active in this compartment [27,28].

Conflicting data have been published concerning Snail1 expression in cancer cells and non-malignant epithelium. While Franci et al. found the protein only in carcinoma cells [16], Bezdekova et al. and others found Snail1 expression in normal epithelium [7,29]. In a previous study with a much smaller cohort (n=10), we were unable to detect Snail1 mRNA expression in normal colonic tissue [21]. However, in this present analysis Snail1 protein expression was also sporadically detected in single cells in the normal colonic tissue, located at the base of crypts. Colonic epithelial stem cells are also believed to be located at the crypt base [30]. Recently, a number of studies have provided evidence that Snail1 is involved in the preservation of stem cell function [31-34]. Whether Snail1 is involved in stem cell functions or cell renewal in colon epithelium are questions we can only speculate about.

Analyses of Snail1 gene expression in different types of human tumours indicate that Snail1 is associated with invasion, secondary metastasis and poor prognosis [35-37]. In our present study, Snail1 expression was detected in 76% of the CRC, similar to previously published expression rates in CRC tissues [13,16]. Downregulation of E-cadherin was observed in 87% of the CRC. The percentage of immunoreactive cells in the samples was variable and heterogeneous for both Snail1 and E-cadherin expression. Interestingly, a significant correlation between Snail1 expression and E-cadherin loss was not detectable. However, we detected a significant correlation between the expression of Snail1 in the tumour and Snail1 expression in the corresponding lymph node metastasis.

We observed significantly elevated Snail1 expression in the tumour centre of small (T1 and T2) compared to advanced tumours (T3 and 4). This could be attributed to transient Snail1 activation [6,38] in the tumour centre of T1 and T2 tumours. There was no difference in Snail1 expression between the T-stages at the invasion front. Snail1 expression at the invasion front was not elevated compared to the tumour centre. Likewise, Snail1 expression was not correlated with histopathological characteristics, such as advanced dedifferentiation (grading) or lymphatic dissemination (N-stage). Interestingly, Franci et al. found higher Snail1 expression at the invasion front of CRC, associated with a significant negative prognostic impact on stage II colon tumours [16]. We noticed a trend between Snail1 expression at the invasion front and loss of E-cadherin in the corresponding lymph node metastasis. Furthermore, Snail1 positive tumours were significantly correlated with Snail1 positive lymph node metastases.

The expression of Snail1 in CRC shows variation in the literature. There is evidence that cells from different tumour compartments interact and thus influence the expression of different oncoproteins. This might explain the observed difference in Snail1 expression between the tumour centre, invasion front and microenvironment. Brabletz et al. found [39] beta-catenin overexpression at the invasion front of CRC. In contrast, cells in the tumour centre often showed no nuclear beta-catenin staining. They postulated that regulatory events in the tumour itself could lead to a different distribution of this oncoprotein. It is possible that surrounding tissue at the invasion front can influence tumour cells, leading to nuclear translocation of beta-catenin, where it may play a direct role in tumour invasion processes [39]. Snail1 is postulated to activate EMT pathways like Wnt signalling by binding to beta-catenin, thereby establishing a positive feedback loop for Wnt-dependent transcription [40]. Thus, Wnt signalling and Snail1-dependent induction of EMT might be interconnected by multiple positive loops, possibly adding to the robustness of both signalling systems. There is evidence for a close relationship between both pathways in vivo, so the loss of E-cadherin could be attributed to the effects of other EMT pathways, perhaps initially triggered by Snail1 activation [10,18].

Becker et al. studied the expression of Snail1 in adenocarcinomas of the upper gastrointestinal tract and found no evidence of any significant association with clinical and pathological parameters [19]. In addition, the same authors detected an association of Snail1 expression with tumour grade in endometrial carcinomas [41] and with overall survival in ovarian carcinomas [42].

In our study, neither Snail1 nor E-cadherin expression seemed to have an effect on overall survival. Since the association between age and overall survival was very strong in this study, the lack of data on disease-specific survival may have led us to underestimate any effects of Snail1 on disease outcome.

Furthermore, we found no evidence of any association of Snail1 with clinicopathological parameters (N-stage, grading, age or sex), with the exception of the significantly elevated Snail1 expression in the tumour centre of small (T1 and T2) compared to advanced (T3 and T4) tumours.

The tumour location, assessed according to the International Classification of Diseases (ICD-10) as endorsed by the WHO, was correlated with the expression profiles of Snail1 and E-cadherin. However, there was no difference in Snail1 expression between rectal, left or right colon cancers.

In conclusion, Snail1 expression was detectable in most of the CRC. Our study indicates that Snail1 expression does not seem to be associated with clinical and pathological data or with overall survival in CRC, even though we cannot rule out an influence on disease-specific survival. Further investigation to assess the relationship between Snail1 and other EMT markers and its relevance in the progression of CRC might be beneficial.

## Conclusion

Snail1 expression was detectable in most of the CRC but showed no significant association with E-cadherin loss, clinical pathological characteristics or overall survival. The observed loss of E-cadherin could be explained by effects of other important EMT pathways, such as the Wnt-signalling cascade.

## Competing interests

There are no financial or other relationships which might lead to a conflict of interest.

## Authors’ contributions

FK Made substantial contributions to conception and design of the manuscript, was involved in drafting the manuscript and revising it critically for important intellectual content. G F Shared first authorship. Acquisition of data has been involved in drafting the manuscript and revising it critically for important intellectual content, carried out the immunoassays. SEB analysis and interpretation of immunochemistry data; has been involved in drafting the manuscript or revising it critically for important intellectual content. LD acquisition of data, carried out the immunoassays. AMR acquisition of data analysis and interpretation of immunochemistry data. DH participated in the design of the study and performed the statistical analysis. DV has been involved in drafting the manuscript and revising it critically for important intellectual content; has given final approval of the version to be published.

NHS has made substantial contributions to conception and design, has given final approval of the version to be published. WTK has made substantial contributions to conception and design; has given final approval of the version to be published. All authors read and approved the final manuscript.

## Authors’ information

Feride Kroepil and Georg Fluegen are shared first authors.

## Pre-publication history

The pre-publication history for this paper can be accessed here:

http://www.biomedcentral.com/1471-2407/13/145/prepub
